# Subclinical Left Ventricular Systolic Dysfunction in HIV Patients: Prevalence and Associations with Carotid Atherosclerosis and Increased Adiposity

**DOI:** 10.3390/jcm11071804

**Published:** 2022-03-24

**Authors:** Eleni Athanasiadi, Maria Bonou, Dimitrios Basoulis, Chris J. Kapelios, Constantina Masoura, Marina Skouloudi, Sophie Mavrogeni, Constantina Aggeli, Mina Psichogiou, John Barbetseas

**Affiliations:** 1 Department of Cardiology, Laiko General Hospital, 115 27 Athens, Greece; eleni-athan@hotmail.com (E.A.); bonou.maria@yahoo.com (M.B.); kmasoura@gmail.com (C.M.); marinoula20@hotmail.com (M.S.); jbnv@otenet.gr (J.B.); 2 First Department of Internal Medicine, Laiko General Hospital, School of Medicine, National and Kapodistrian University of Athens, 115 27 Athens, Greece; dimitris.bassoulis@gmail.com (D.B.); mpsichog@med.uoa.gr (M.P.); 3 Department of Cardiology, Onassis Cardiac Surgery Center, 176 74 Athens, Greece; sophie.mavrogeni@gmail.com; 4 First Department of Cardiology, Hippokration General Hospital, School of Medicine, National and Kapodistrian University of Athens, 115 27 Athens, Greece; dina.aggeli@gmail.com

**Keywords:** HIV infection, left ventricular dysfunction, speckle tracking, echocardiography, carotid atherosclerosis, inflammation

## Abstract

Background: Human immunodeficiency virus (HIV) is mainly detected in young, otherwise healthy, individuals. Cardiomyopathy and peripheral artery disease affecting these patients appears to be multifactorial. Prompt and potentially more effective implementation of therapeutic measures could be enabled by pre-symptomatic diagnosis of myocardial dysfunction and peripheral artery damage. However, limited data is available to date on this specific topic. Μethods: We investigated the association between global longitudinal strain (GLS), an established index of subclinical left ventricular systolic dysfunction (LVSD) assessed by two-dimensional speckle-tracking echocardiography, and: (a) patient history; (b) demographic and clinical baseline characteristics; (c) carotid intima-media thickness (IMT) and the presence of carotid atherosclerotic plaque(s), measured by ultrasonography; (d) temperature difference (ΔT) along each carotid artery, measured by microwave radiometry; and (e) basic blood panel measurements, including high-sensitivity troponin-T (hsTnT) and NT-proBNP in people living with HIV (PLWH) and no history of cardiovascular disease. Results: We prospectively enrolled 103 consecutive PLWH (95% male, age 47 ± 11 years, anti-retroviral therapy 100%) and 52 age- and sex-matched controls. PLWH had a significantly higher relative wall thickness (0.38 ± 0.08 vs. 0.36 ± 0.04, *p* = 0.048), and higher rate of LVSD (34% vs. 15.4%, *p* = 0.015), and carotid artery atherosclerosis (28% vs. 6%, *p* = 0.001) compared with controls. Among PLWH, LVSD was independently associated with the presence of carotid atherosclerosis (adj. OR:3.09; 95%CI:1.10–8.67, *p* = 0.032) and BMI (1.15; 1.03–1.29, *p* = 0.017), while a trend for association between LVSD and left ventricular hypertrophy was also noted (3.12; 0.73–13.33, *p* = 0.124). No differences were seen in microwave radiometry parameters, NT-proBNP, hs-TnT and c-reactive protein between PLWH with and without LVSD. Conclusions: Subclinical LVSD and carotid atherosclerosis were significantly more frequent in PLWH compared to a group of healthy individuals, implying a possible link between HIV infection and these two pathological processes. Carotid atherosclerosis and increased adiposity were independently associated with impaired GLS in HIV-infected individuals.

## 1. Introduction

The advent of antiretroviral therapy (ART), as well as the early diagnosis of HIV, has extended the life expectancy of people living with HIV (PLWH), which now approaches that of the general population [1,2]. A main source of morbidity and mortality in this population is cardiovascular disease (CVD), which is responsible for up to 11% of deaths and presents mostly as acute coronary syndromes (ACS) and HIV-associated cardiomyopathy (HIVAC) [3,4]. The pathophysiology behind accelerated coronary artery disease (CAD) seems to be related to unstable, non-calcified atherosclerotic plaques, platelet reactivity, pro-coagulant state, and inflammation, as well as to the higher prevalence of common CVD risk factors, such as hypertension, dyslipidemia, diabetes, and tobacco use [5,6,7,8,9,10,11]. As for HIVAC and heart failure (HF), they are considered to be the end result of cardiomyocyte inflammation and apoptosis (from HIV itself but also from other opportunistic infections), immune system dysregulation with the augmented expression of autoantibodies (caused by traditional cardiac risk factors and ART), and micronutrient deficiency [12,13].

In the era of ART, cardiomyopathy is mainly expressed as subclinical diastolic dysfunction or impaired cardiac strain. To date, conventional echocardiography continues to be the basis for the diagnosis of HF in this population. The use of novel imaging techniques, such as speckle tracking and global longitudinal strain (GLS), to detect early myocardial dysfunction before the presentation of symptoms can help timely diagnosis and decisive treatment of this vulnerable group. As for the detection of subclinical atherosclerosis, novel techniques such as coronary artery calcium scoring and computed tomography angiography are not only expensive, but also expose the examinee to excessive radiation [14]. Novel, non-invasive and inexpensive techniques need to be devised and tested. Microwave radiometry (MWR) is a new, time-saving and inexpensive technique, which may be helpful for detecting subclinical atherosclerosis, especially vulnerable atherosclerotic plaques, via assessing thermal heterogeneity of carotid arteries [15]. This method seems to be able to detect the presence of CAD in patients with diabetes type 2 and to predict major CV events during 1-year follow-up in patients with known CAD [16].

Due to the limited data on speckled tracking study in HIV patients, there is a need to understand the prevalence of LVSD and its correlates in this cohort of patients. Interestingly, a strong relationship between carotid atherosclerosis with subclinical LVSD and clinical HF in the general population has been recently reported, but there are no reports in PLWH [17,18]. The objective of this cross-sectional study was to investigate: (a) the prevalence of left ventricular systolic dysfunction (LVSD) assessed by two-dimensional (2D) speckle-tracking echocardiography; (b) the relationship of LVSD with carotid atherosclerosis and temperature heterogeneity, measured by carotid ultrasound and MWR, respectively; and (c) the associations between LVSD and factors of patient history, clinical status and vascular studies in unselected, asymptomatic PLWH free of known CVD.

## 2. Materials and Methods

### 2.1. Methods

This cross-sectional study complies with the Declaration of Helsinki and was approved by the institutional review board of Laiko General Hospital, a university tertiary hospital in Athens. All participants provided written informed consent.

### 2.2. Study Population

One hundred and three consecutive consenting PLWH who were followed in the outpatient Infectious Diseases Unit of the First Department of Internal Medicine were recruited. Exclusion criteria were: (1) age <18 years; (2) history of coronary heart disease, valvular disease, cardiomyopathy; (3) significant systolic dysfunction of the LV defined as EF <50%; (4) active injection drug use; and (5) co-infection with hepatitis B or C. A control group of fifty-two healthy individuals (2:1 nearest neighbor matching), matched for age and sex served as the control group to assess for the differential prevalence of LVSD and carotid atherosclerosis in PLWH vs. people living without HIV.

### 2.3. Study Design

All study participants underwent complete transthoracic echocardiography (TTE), including analysis with 2D speckle tracking and carotid ultrasound for carotid intima-media thickness (IMT) and the presence of carotid atheromatic plaque(s), and MWR for temperature difference (ΔT) measurement along each carotid artery and basic blood panel measurements, including c-reactive protein (CRP), high-sensitivity troponin-T (hs-TnT), and NT-pro-BNP.

### 2.4. Study Procedures and Data Collection

Demographic characteristics, detailed medical history, including classical CVD risk factors, drug regimen and doses were recorded at baseline. The patients underwent thorough laboratory examination. Additionally, CD4 count, receipt of antiretroviral medications and history of antiretroviral exposure to each agent, nadir CD4 count and disease duration were also recorded.

### 2.5. Echocardiography

All patients underwent comprehensive transthoracic (two-dimensional, Doppler) and speckle-tracking echocardiography using the Vivid-7 ultrasound system equipped with a 2.5–3.5 MHz probe, and EchoPAC software (General Electric Healthcare, Chicago, IL, USA), respectively. Standard echocardiographic measurements were assessed in accordance with current guidelines [19]. Left ventricular ejection fraction and left atrial volumes were obtained from 4- and 2-chamber apical views and were calculated using the modified Simpson’s biplane method. The volume of the left atrium volume was then indexed for body surface area. Peak early transmitral flow velocity (E-wave), recorded by pulsed-wave Doppler and early mitral annular tissue velocity (e’-wave), assessed by tissue Doppler, were obtained from the apical 4-chamber view. Diastolic dysfunction was defined as the presence of one of the following: (a) septal e’ velocity < 7 cm/s or lateral wall e’ velocity < 10 cm/s; or (b) evidence of chronically elevated LV filling pressure or LV hypertrophy, including left atrial volume index > 34 mL/m^2^, LV mass index > 95/m^2^ in women or >115 g/m^2^ in men, or relative wall thickness >0.42 [20,21]. We used the average values of five beats for analysis. The primary study variable was LVSD, defined as a value of GLS less negative than −18.0%, as previously used [22]. GLS, an early marker of myocardial dysfunction, was measured offline with speckle-tracking echocardiography and was defined as the mean peak longitudinal strain of the 17 segments of the LV measured in the apical 4-, 3- and 2-chamber views. All strain values were performed by an experienced cardiologist (MB), blinded to the echocardiographic images.

### 2.6. Carotid Ultrasound

We used ultrasonography imaging with UltraSound GE Vivid-7 Pro (General Electric Healthcare, Chicago, IL, USA) to reveal the presence of atheromatic plaques in the common carotid artery, the carotid sinus and the internal carotid artery. A plaque was defined as a bulging to the lumen intima-medial thickness (IMT) greater than 1.5 mm, or a local increase in the IMT of more than 50% compared to the adjacent vessel wall, according to international guidelines [23]. IMT was considered abnormal when its value was above the 90th percentile of the age limit or/and above the high-risk cut-off value of 0.9 mm [24]. All exams were performed by a single operator (CM).

### 2.7. Radiometry

The MWR measurements were performed with the RTM 01 RES microwave computer-based system (Bolton, UK) by measuring the temperature of internal tissues at microwave frequencies. Segments of 20 mm in length were analyzed by placing the microwave antenna of the device at a 90° angle to the skin, starting from the proximal common carotid artery, and moving distally to avoid overlapping or missing areas. Carotid temperature measurements were performed three times on each segment. ΔT for each carotid artery was defined as the temperature of the segment with the highest temperature minus the lowest temperature for each carotid. In the analysis, “ΔTmax” was defined as the maximum ΔT value of both carotid arteries. All exams were performed by a single operator (EA).

### 2.8. Statistical Analysis

Categorical variables are presented as % and were compared using the chi-square test. Normally distributed continuous variables, as assessed by the Kolmogorov–Smirnov test, are presented as means ± standard deviation (SD) and were compared by using the Student’s t-test between groups. Non-normally distributed continuous variables are presented as medians (1st–3rd quartile) and were compared using the Mann–Whitney test. To assess for associations between the presence of LVSD and other study variables, logistic regression univariable and multivariable analyses were utilized. Covariates that were significant in the univariable analysis were entered into the multivariable model. Associations are presented as hazard ratio (HR) and two-sided 95% confidence intervals (CI). All *p* values were two-sided and a *p*-value <0.05 was considered statistically significant for all analyses. The Statistical Package for the Social Sciences (IBM SPSS software version 24.0 for Windows, Armonk, NY, USA) was used for the analysis.

## 3. Results

### 3.1. Comparisons between PLWH and Controls

#### 3.1.1. Echocardiography

One hundred and three consecutive PLWH were enrolled between September 2018 and June 2020. Mean age of the participants was 47 ± 11 years, 95% were male, 46% were active smokers, and mean HIV duration was 8.1 ± 6.0 years. Baseline characteristics of the study cohort are presented in Table 1. Mean age of the healthy individuals of the control group was 46 ± 8 years, 96% were male and 37% were active smokers. Their baseline characteristics are also presented in Table 1. The PLWH and control group did not differ significantly with regard to BMI, smoking status, or prevalence of comorbidities, such as hypertension, dyslipidemia and diabetes.

PLWH were detected to have smaller left ventricles (end-diastolic diameter of 48.1 ± 3.7 vs 49.4 ± 2.9 mm, *p* = 0.014) with thicker walls (relative wall thickness 0.38 ± 0.08 vs. 0.36 ± 0.04, *p* = 0.048) compared with controls. PLWH presented significantly more often with LVSD compared with controls (34% vs. 15.4%, *p* = 0.015), while no significant difference in the prevalence of diastolic dysfunction was noted. All comparisons regarding echocardiography between PLWH and controls are depicted in Table 1.

#### 3.1.2. Carotid Ultrasound

The results of the baseline vascular disease studies among PLWH and controls are demonstrated in Table 2. IMT higher than the cut-off of 0.9 mm was present in either carotid artery in 66% of PLWH vs. 42% of controls (*p* = 0.005). Carotid artery atherosclerosis was diagnosed in 28% of PLWH vs. 6% of controls (*p* = 0.001).

#### 3.1.3. Microwave Radiometry

The mean value of ΔΤ in the left carotid artery was 0.4 ± 0.3 vs. 0.5 ± 0.2 °C (*p* = 0.12) and in the right carotid artery 0.4 ± 0.2 vs. 0.5 ± 0.2 °C (*p* = 0.06) for PLWH vs. controls, respectively, whereas ΔΤmax was 0.5 ± 0.2 vs. 0.6 ± 0.2 °C (*p* = 0.37). The results of the baseline microwave radiometry study between PLWH and controls are demonstrated in Table 2.

### 3.2. Comparisons among PLWH

Within the PLWH cohort, participants with LVSD (*n* = 35) had higher BMI (28 ± 6 kg/m^2^ vs. 25 ± 3 kg/m^2^, *p* = 0.002) and larger waist circumference (103 ± 14 cm vs. 92 ± 10 cm, *p* < 0.001) compared to patients without LVSD (*n* = 78) (Table 3). Furthermore, PLWH with LVSD had thicker interventricular (9.9 ± 1.5 mm vs. 9.0 ± 1.2 mm, *p* = 0.001) and posterior left ventricular walls (9.6 ± 1.5 mm vs. 8.7 ± 1.6 mm, *p* = 0.006), higher relative wall thickness (0.40 ± 0.08 vs. 0.36 ± 0.07, *p* = 0.018), higher left ventricular mass index (80.1 ± 16.8 vs. 73.9 ± 12.6 gr/m^2^, *p* = 0.043), and a higher prevalence of left ventricular hypertrophy (26% vs. 8%, *p* = 0.015) and diastolic dysfunction (51% vs. 22%, *p* = 0.002) compared with PLWH without LVSD (Table 3). HIV-related parameters, CRP, hs-TnT, or NT-proBNP, did not differ between PLWH with and without LVSD (Table 3). Interestingly, though, PLWH with LVSD had a higher prevalence of carotid atherosclerosis compared with PLWH without LVSD (47% vs. 19%, *p* = 0.005), whereas no difference was noted between PLWH with and without LVSD regarding microwave radiometry parameters (Table 3).

In multivariable logistic regression analysis, the parameters that were significantly and independently associated with LVSD were the presence of carotid atherosclerosis (adj. OR: 3.09; 95%CI: 1.10–8.67, *p* = 0.032) and BMI (adj. OR: 1.15; 95% CI: 1.03–1.29, *p* = 0.017), while a trend for the association between LVSD and left ventricular hypertrophy was also noted (adj. OR: 3.12; 95% CI: 0.73–13.33, *p* = 0.124) (Table 4). The association between LVSD and diastolic dysfunction was significant in univariable analysis but was rendered insignificant after adjusting for the other covariates (adj. OR: 1.19; 95% CI: 0.32–4.38, *p* = 0.793).

The study population was further divided into four weight groups: normal weight (BMI 18.5–24.9 kg/m^2^, Ν = 49); overweight (BMI 25–29.9 kg/m^2^, Ν = 39); moderately obese (BMI 30–34.9 kg/m^2^, Ν = 11); and severely obese (BMI ≥ 35 kg/m^2^, Ν = 4). The prevalence of LVSD was 33%, 46%, 64%, and 100% among patients in the 1st, 2nd, 3rd, and 4th BMI groups, respectively (*p* = 0.024). A significant increase in LVSD with each increase in BMI category was observed in the univariate regression analysis (OR: 2.2; 95% CI: 1.3–3.8, *p* = 0.005).

## 4. Discussion

In this study, performed in relatively young PLWH, without known CVD, LVSD was detected in approximately 1/3 of PLWH. LVSD was significantly more common among PLWH compared with a group of individuals matched for age and sex, thus underpinning a possible association between HIV infection and LVSD. Among the PLWH, LVSD was significantly linked with increased adiposity and the presence of carotid atherosclerosis. Furthermore, abnormal carotid IMT and the presence of carotid atherosclerosis was highly prevalent in this population.

### 4.1. Echocardiography

HIV infection is known to be associated with imaging findings of cardiac dysregulation even before the onset of symptoms of HF. In accordance with previous findings, our study showed that 34% of the studied population had left ventricular systolic dysfunction, as estimated by GLS. In keeping with this, Buggey et al., also found that HIV was associated with increased left ventricular mass index, decreased LV GLS, and higher odds of diastolic dysfunction, underscoring the contribution of an inflammatory process to the development of HF with preserved ejection fraction among PLWH [25]. In addition, Sukru et al. recorded that PLWH had lower GLS compared with healthy individuals, despite normal LV systolic function and no cardiac symptoms [26]. Rodrigues et al. reported similar results by showing lower median GLS in both untreated (−17.70%) and treated with non-nucleoside reverse transcriptase (−18.47%) or protease inhibitor (−18.27%) PLWH compared with controls (−20.77%) [27]. In our study, the mean value of GLS was −19.3%, which is lower in absolute value than the one previously reported among healthy controls tested with the same vendor, but higher than ones reported with other vendors [28], underlining the variability of GLS values according to vendor used [28]. This may have significant implications in the cut-off selection to define LVSD. In our study, we defined impaired GLS as ≥−18.0%, based on the fact that our study population comprised a group of relatively young adults that were free of known CVD [22]. Importantly, GLS evaluation using magnetic resonance imaging in asymptomatic PLWH also revealed the presence of subclinical systolic dysfunction [29,30].

In our study, LVH was associated with LVSD in the univariate regression analysis, although this relationship was rendered insignificant after adjusting for confounders. In line with this, Isast et al. found a higher prevalence of LVH in asymptomatic HIV-infected patients (28.6%) compared with a general European population of similar characteristics (12%) [31]. Hsue et al. also reported HIV infection to be independently associated with a higher LV mass index in both unadjusted and adjusted analyses [32]. Apart from traditional risk factors for LVH (age, hypertension, diabetes mellitus), it seems that ART or even direct inflammation of the myocardium from HIV can be related to increased myocardial mass in PLWH [33,34].

The prevalence of obesity and visceral adiposity is increased among PLWH as a result of traditional contributors (Western diet, sedentary lifestyle) as well as ART and HIV infection, through chronic inflammation, metabolic dysregulation, and immune activation [35,36]. In our study LVSD was not only significantly associated with BMI, but a further impairment of LV systolic function as the BMI increased was noted. Obesity is already known to be related to cardiac dysregulation, mainly through chronic volume overload, LV hypertrophy, and LV diastolic dysfunction [37,38]. While overt systolic dysfunction with reduced EF is less common in obese individuals, asymptomatic LVSD is increasingly reported in patients with preserved EF through novel techniques, such as speckle tracking [38,39,40]. While the precise mechanism is not yet fully elucidated, it seems to be a combination of intracellular lipid accumulation and lipotoxicity with subsequent cardiomyocyte apoptosis, insulin resistance, neuroendocrine activation, and the promotion of other risk factors such as hypertension, dyslipidemia, and diabetes mellitus [39,41]. Nevertheless, in our study, PLWH had a higher prevalence of LVSD compared to controls with matched BMI, underscoring a link between HIV infection and subclinical LVSD.

In addition, diastolic dysfunction was detected in 32% of the relatively young cohort of PLWH, and its prevalence was statistically higher in PLWH with LVSD than in those without systolic impairment. This is in line with previous studies showing high rates of diastolic dysfunction among PLWH, and being more pronounced in younger subjects [21,42]. In a study by Oursler et al., diastolic dysfunction was present in 38% of HIV patients and associated with reduced cardiorespiratory fitness, measured by oxygen consumption at peak exercise, highlighting the importance of recognizing diastolic dysfunction in individuals living with HIV [43].

Furthermore, an intriguing finding is that LVSD was strongly associated with the presence of carotid artery atherosclerosis in asymptomatic well-treated PLWH. The association of both IMT and carotid plaque burden with systolic and diastolic dysfunction, measured by transthoracic echocardiography, as well as with HF hospitalizations, has been reported in healthy individuals after adjustment for traditional risk factors, with the subjects at the upper quartile of IMT being at higher risk [17,44]. Additionally, in a recent study IMT was an independent risk factor of subclinical LVSD in the general population, as assessed by speckle tracking [18]. Furthermore, adverse changes in carotid structure, as expressed with increased IMT, were inversely correlated with LV GLS in obese patients with type 2 diabetes mellitus [45]. To our knowledge, our study is the first to demonstrate an adverse relationship of carotid atherosclerosis with subclinical LVSD among PLWH. Nevertheless, this finding was obtained in a small cohort and further studies are warranted to confirm this association. Although the underlying mechanisms of this link need to be clarified, there may be several explanations: (i) PLWH, despite ART, have evidence of HIV-related persistent systemic low-level inflammation, potentially resulting in both functional alterations of the LV and carotid atherosclerosis progression in this population [46]; (ii) increased IMT is associated with a greater likelihood of impaired coronary flow reserve due to epicardial vessel stenosis or/and coronary microvasculature dysfunction, which may cause LVSD [47]; and (iii) the higher incidence of comorbidities such as obesity, diabetes mellitus and mainly hypertension among PLWH, may accelerate vascular structural changes, increase IMT and arterial stiffening, and thus impair LV systolic function [5,17]. 

Finally, no association between LVSD, serum levels of NT-proBNP, and high-sensitivity cardiac troponin were demonstrated in our study. Our understanding is that GLS is extremely sensitive in detecting slight reductions in LV systolic function. In cases where there is damage to cardiomyocytes (e.g., hypertrophic cardiomyopathy, cardiotoxicity) accompanied by an increase in troponin levels, there is also typically impairment in GLS [48,49]. In other cases, though, no association has been noted between troponin leak and GLS, even when the mechanism of LV dysfunction is coronary artery disease or inflammation [50,51,52]. Based on the above, one could advocate that GLS is affected earlier in the sequalae of the cardiomyopathic or inflammatory process, prior to the point when serum markers of cardiomyocyte damage or dysfunction (troponin, natriuretic peptides) are found to be elevated.

### 4.2. Carotid Ultrasound

Consistent with previous studies, PLWH showed an increased incidence of carotid atherosclerosis with abnormal IMT (taking into account both age-adjusted IMT and the cut-off of 0.9 mm). Psichogiou et al. came to similar results by exhibiting an increased risk of carotid atherosclerosis, especially at the site of the left carotid bulb in asymptomatic PLWH, as well as rapid annual progression compared with non-HIV individuals with several risk factors [53]. In addition, by the PET-CT quantification of 18-Fluorodeoxyglucose (18-FDG), Yarasheski et al. revealed greater right and left carotid 18-FDG uptake in HIV-infected adults in comparison with HIV-negative participants, consistent with vascular inflammation and early atherosclerosis in this population [54].

### 4.3. Microwave Radiometry

Cardiovascular disease is a main cause of non-HIV-related morbidity and mortality in PLWH. Atherosclerosis in this population is mainly related to the presence of unstable atherosclerotic plaques, non-calcified, with a necrotic lipid-rich core and a higher risk of rupture. These vulnerable plaques are associated with higher degrees of inflammation due to immune activation [55]. MWR is a novel, non-invasive method that has been shown to reveal vulnerable plaques and detect inflammation through relative differences of temperature in carotid arteries, with an accuracy for temperature measurements of ±0.20 °C, and with promising results in previous studies [15,16,56,57]. Nonetheless, in the present study, there was no difference in MWR parameters between PLWH and controls or between PLWH with and without LVSD. Although ΔΤmax has been associated with carotid inflammation as well as major cardiovascular events in patients with CAD, no study to date has correlated it with LVSD. Thus, our findings cannot be considered as unexpected. Their clinical relevance, though, needs further investigation in a subset of PLWH with no or suboptimal ART, given that (a) MWR is designed to detect active inflammation, and (b) that our PLWH cohort had been virally suppressed under ART for a long period prior to the study.

### 4.4. Limitations

Due to the single-center design and the relatively small study population, generalization of the results should be applied with caution before confirmation is available from larger population analyses. Furthermore, we did not measure markers of endothelial dysfunction that could potentially explain a potential contribution of HIV infection to the subclinical changes in LV and carotid arteries; thus, causation cannot be inferred from our study. Moreover, although a comprehensive multivariable analysis was performed, we cannot exclude the contribution of unidentified confounders to the reported associations.

## 5. Conclusions

In conclusion, pre-symptomatic LVSD is a common finding in young PLWH, free of CVD, underlining that chronic inflammatory stimulation can potentially play an important role as a mechanistic pathway. Carotid atherosclerosis and increased adiposity were associated with the presence of LVSD, underscoring the possible contribution of atherosclerosis to the development of impaired systolic myocardial function. Further investigation is warranted to confirm these findings and identify patients at high risk, for whom early screening and preventive interventions are required, as well as to determine possible associations between risk markers identified in the present analysis and long-term outcomes.

## Figures and Tables

**Table 1:** Demographic and clinical characteristics of PLWH and controls.

Characteristics	PLWH (N = 103)	Controls (Ν = 52)	*p*
Age, years	47 ± 11	46 ± 8	0.60
Male Gender, %	95	96	1.00
Body mass index, kg/m^2^	26 ± 4	27 ± 3	0.51
Waist, cm	96 ± 13	-	-
Smoking, %			0.45
Active	46	37	
Former	17	19	
Hypertension, %	23	27	0.62
Dyslipidemia, %	43	40	0.78
Diabetes, %	6	4	0.60
**Laboratory findings**			
Total cholesterol, mg/dL	186 ± 40	-	-
Low-density lipoprotein, mg/dL	115 ± 34	-	-
High-density lipoprotein, mg/dL	45 ± 12	-	-
Triglycerides, mg/dL	130 ± 71	-	-
Hgb, gr/dL	14.9 ± 1.3	-	-
Urea, mg/dL	35 ± 10	-	-
Creatinine, mg/dL	1.0 ± 0.2	-	-
High-sensitivity troponin-T, pg/mL	6.2 ± 4.8	-	-
NT-proBNP, pg/mL	26 (14, 53)	-	-
**HIV clinical factors**			
HIV infection duration, years	8.1 ± 6.0	-	-
Median CD4 T-cell count (IQR)	656 (451, 853)	-	-
Median nadir CD4 T-cell count (IQR)	281 (101, 420)	-	-
HAART experienced, %	100	-	-
**Echocardiography**			
Interventricular septum, mm	9.3 ± 1.4	8.9 ± 1.0	0.055
Posterior wall, mm	9.0 ± 1.6	8.8 ± 1.0	0.37
Left-ventricular end-diastole, mm	48.1 ± 3.7	49.4 ± 2.9	0.014
Left ventricular hypertrophy, %	14	3.8	0.051
Relative wall thickness	0.38 ± 0.08	0.36 ± 0.04	0.048
Left ventricular mass index, gr/m^2^	76.1 ± 14.4	74.8 ± 12.8	0.591
Left atrium volume index, ml/m^2^	25.0 ± 6.0	26.1 ± 5.4	0.248
Left ventricular ejection fraction, %	60.1 ± 4.0	60.2 ± 2.5	0.784
Diastolic dysfunction, %	32	19	0.093
Global longitudinal strain, %	−19.3 ± 3.0	−19.2 ± 1.4	0.832
Left ventricular systolic dysfunction, %	34	15.4	0.015

**Table 2:** Baseline assessment of vascular markers in PLWH and controls.

Variable	HIV Infected (N = 103)	Controls (Ν = 52)	*p*
**Carotid ultrasound**			
Left common carotid artery intima media thickness, mm	7.5 ± 3.0	6.8 ± 1.9	0.07
Left carotid artery bulb intima media thickness, mm	10.4 ± 4.3	9.3 ± 3.5	0.02
Left internal carotid artery intima media thickness, mm	8.0 ± 4.2	6.8 ± 24	0.04
Right common carotid artery intima media thickness, mm	7.0 ± 1.8	6. 9 ± 1.9	0.69
Right carotid artery bulb intima media thickness, mm	11.2 ± 5.6	9.1 ± 3.0	0.004
Right internal carotid artery intima media thickness, mm	8.7 ± 5.3	6.8 ± 2.4	0.004
Abnormal for age limit right carotid artery intima media thickness, %	47	46	0.36
Abnormal for age limit left carotid artery intima media thickness, %	49	42	0.41
Any carotid artery abnormal for age limit intima media thickness, %	61	62	0.89
Right carotid artery intima media thickness >0.9 mm, %	54	45	0.27
Left carotid artery intima media thickness >0.9 mm, %	49	39	0.21
Any carotid artery intima media thickness >0.9 mm, %	66	42	0.005
Right carotid artery atherosclerosis %	19	4	0.012
Left carotid artery atherosclerosis, %	16	2	0.011
Any carotid artery atherosclerosis, %	28	6	0.001
**Microwave Radiometry**			
Maximum temperature of left carotid (°C)	35.2 ± 0.6	35.4 ± 0.7	0.04
Minimum temperature of left carotid (°C)	34.8 ± 0.7	34.9 ± 0.8	0.14
ΔT left (°C)	0.4 ± 0.3	0.5 ± 0.2	0.12
Maximum temperature of right carotid (°C)	35.3 ± 0.7	35.2 ± 0.7	0.19
Minimum temperature of right carotid (°C)	34.9 ± 0.8	34.7 ± 0.8	0.07
ΔT right (°C)	0.4 ± 0.2	0.5 ± 0.2	0.06
ΔΤ max (°C)	0.5 ± 0.2	0.6 ± 0.2	0.37

**Table 3:** Characteristics of PLWH with and without early left ventricular systolic dysfunction.

Characteristics	LVSD (N = 35)	No LVSD (Ν = 68)	*p*-Value
Age, years	48 ± 11	46 ± 11	0.37
Male Gender, %	97	94	0.5
Body mass index, kg/m^2^	28 ± 6	25 ± 3	0.002
Waist, cm	103 ± 14	92 ± 10	<0.001
Smoking, %			0.89
Active	43	47	
Former	14	18	
Hypertension, %	29	31	0.36
Dyslipidemia, %	51	38	0.20
Diabetes, %	2.9	7.4	0.36
**Laboratory findings**			
Total cholesterol, mg/dL	183 ±37	187 ± 42	0.64
Low-density lipoprotein, mg/dL	112 ± 33	116 ± 35	0.61
High-density lipoprotein, mg/dL	43 ± 14	46 ± 11	0.14
Triglycerides, mg/dL	142 ± 86	125 ± 61	0.25
Hgb, g/dL	14.8 ± 1.4	14.9 ± 1.3	0.56
Urea, mg/dL	35 ± 10	35 ± 10	0.88
Creatinine, mg/dL	1.0 ± 0.2	1.0 ± 0.2	0.43
High-sensitivity troponin-T, pg/mL	6.5 ± 4.8	6.1 ± 4.8	0.71
Median NT-proBNP, pg/mL	29 (17, 55)	25 (13, 52)	0.56
C-reactive protein (mg/L)	3.1 ± 4.1	2.7 ± 3.8	0.52
**HIV clinical factors**			
HIV infection duration, years	9.2 ± 6.7	7.5 ± 5.5	0.17
Median CD4 T-cell count (IQR)	693 (487, 1030)	652 (442, 840)	0.63
Median nadir CD4 T-cell count (IQR)	269 (78, 396)	305 (104, 435)	0.45
HAART experienced, %	100	100	1.00
**Echocardiography**			
Interventricular septum. mm	9.9 ± 1.5	9.0 ± 1.2	0.001
Posterior wall, mm	9.6 ± 1.5	8.7 ± 1.6	0.006
Left-ventricular end-diastole, mm	48.3 ± 4.1	48.0 ± 3.5	0.69
Left ventricular hypertrophy	26	8	0.015
Relative wall thickness	0.40 ± 0.08	0.36 ± 0.07	0.018
Left ventricular mass index, gr/m^2^	80.1 ± 16.8	73.9 ± 12.6	0.043
Left atrium volume index, mL/m^2^	35.1 ± 4.3	34.5 ± 4.4	0.45
Diastolic dysfunction, %	51	22	0.002
**Carotid ultrasound**			
Any carotid artery abnormal for age limit intima media thickness, %	67	58	0.41
Any carotid artery intima media thickness >0.9 mm, %	73	63	0.32
Right carotid artery atherosclerosis, %	30	14	0.06
Left carotid artery atherosclerosis, %	20	14	0.43
Any carotid artery atherosclerosis, %	47	19	0.005
**Microwave Radiometry**			
Maximum temperature of left carotid (°)	35.0 ± 0.7	35.3 ± 0.6	0.07
Minimum temperature of left carotid (°)	34.6 ± 0.7	34.8 ± 0.7	0.12
ΔT left (°)	0.4 ± 0.2	0.4 ± 0.3	0.7
Maximum temperature of right carotid (°)	35.2 ± 0.7	35.4 ± 0.7	0.17
Minimum temperature of right carotid (°)	34.8 ± 0.8	35.0 ± 0.7	0.15
ΔT right (°)	0.4 ± 0.2	0.4 ± 0.3	0.39
ΔΤ max (°)	0.5 ± 0.2	0.6 ± 0.3	0.38

**Table 4:** Univariable and multivariable logistic regression analysis for the presence of left ventricular systolic dysfunction among PLWH.

Variable	Univariable Analysis OR (95% CI)	*p*	Multivariable Analysis OR (95% CI)	*p*
Age	1.02 (0.98–1.06)	0.37	-	-
Male gender	2.13 (0.22–19.8)	0.51	-	-
Duration of disease	1.05 (0.98–1.12)	0.17	-	-
Nadir CD4 count	1.000 (0.998–1.001)	0.58	-	-
Hypertension	1.54 (0.60–3.95)	0.37	-	-
Diabetes	0.37 (0.03–3.30)	0.37	-	-
Body mass index	1.17 (1.05–1.30)	0.004	1.15 (1.03–1.29) *	0.017
Waist	1.08 (1.03–1.13)	0.001	-	*-*
HDL	0.97 (0.94–1.01)	0.14	-	*-*
CD4	1.001 (1.000–1.002)	0.16	-	-
High-sensitivity troponin-T	1.016 (0.93–1.11)	0.71	-	-
NT-proBNP	1.004 (0.99–1.01)	0.32	-	-
Intraventricular septum	1.73 (1.21–2.47)	0.002	-	-
Posterior wall	1.60 (1.14–2.25)	0.007	-	-
LVH	4.09 (1.25–13.39)	0.020	3.12 (0.73–13.33) *	0.124
Diastolic dysfunction	3.74 (1.56–8.99)	0.003	1.19 (0.32–4.38)	0.793
Carotid atherosclerosis	3.82 (1.45–10.08)	0.007	3.09 (1.10–8.67)	0.032

* BMI was entered over waist circumference in the multi-variable model as a marker of adiposity and LVH over intraventricular septum and posterior wall thickness.

## Data Availability

The data presented in this study are available on request from the corresponding author. The data are not publicly available due to GDPR Protection Regulation.
